# Cross-Sectional Study Comparing Activities of Daily Living Using Barthel Index Within a Geriatric Population Having Non-Communicable Diseases

**DOI:** 10.7759/cureus.65046

**Published:** 2024-07-21

**Authors:** Manasi Harale, Arun Oommen, Ahsan A Faruqi, Mayank Mundada, Tushar Pancholi, Bhavya Yammanuru, Sree Vidya Yekkaluru, Raju Hansini Reddy, Abishak D Gupta

**Affiliations:** 1 General Medicine, Dr. D Y Patil Medical College, Hospital and Research Centre, Pune, IND

**Keywords:** lifestyle diseases prevention, old age homes, health public, medical comorbidities, barthel’s index of activities of daily living, geriatric medicine

## Abstract

Introduction

Disabilities and non-communicable diseases (NCDs) are prevalent among the elderly, significantly affecting their quality of life. Comprehensive population-based data are essential for effective healthcare planning and rehabilitation. This study aims to determine the prevalence of self-reported disabilities and compare Barthel Index scores among elderly individuals with and without NCDs.

Methods

A cross-sectional study was conducted at Dr. D. Y. Patil Medical College, Pune, involving 102 patients aged 60 years and above. Patients with a history of strokes or limb amputations were excluded. Data on demographics, comorbidities, and functional status were collected using a structured questionnaire designed based on Barthel Index scoring to assess the activities of daily living (ADL).

Results

The study included 102 participants: 58 males (56.9%) and 44 females (43.1%). Age distribution showed 73.5% in the 60-74 age group, 22.5% in the 75-84 age group, and 3.9% in the 85+ age group. Comorbidity data revealed that 37.3% had no comorbidities, 26.4% had one comorbidity, and 36.3% had two or more comorbidities. The mean Barthel Index scores were 87.11 for those without comorbidities, 83.89 for those with one comorbidity, and 82.30 for those with two or more comorbidities. The most affected activities were stair climbing (75.7%), bowel control (48.5%), and mobility (47.1%).

Conclusion

NCDs significantly impact daily activities in the elderly, underscoring the need for targeted healthcare interventions to improve their quality of life. This study highlights the importance of comprehensive care strategies to address the specific needs of elderly patients with comorbidities.

## Introduction

Non-communicable diseases (NCDs), such as diabetes, hypertension, and ischemic heart disease, are major health concerns globally, particularly among the elderly population. According to the World Health Organization (WHO), 15% of the global population is disabled, with a significant proportion being elderly individuals who often suffer from one or more chronic conditions [1]. This demographic trend necessitates a comprehensive understanding of the impact of NCDs on the daily lives of the elderly in order to inform healthcare planning and rehabilitation strategies. Previous studies have highlighted the substantial physical dependency among the elderly due to NCDs. For instance, a study conducted in Uttarakhand, India, found that 28% of the elderly population were physically dependent for their daily activities, with a significant proportion experiencing moderate to severe dependency [2]. This study underscores the urgent need for improved home care and geriatric health services to manage the increasing burden of NCDs [3-6]. Recent studies have also explored the impact of social participation and quality-of-life tools in enhancing community aged care [7], advancements in regenerative medicine, and the biology of aging [8]. Additionally, physical activity has been shown to attenuate total and cardiovascular mortality associated with physical disability among older adults, emphasizing the need for integrated care approaches that include physical activity as a core component [9].

The Barthel Index is a widely used tool to measure performance in activities of daily living (ADL). The values assigned to each item are based on the time and amount of actual physical assistance required if a patient is unable to perform the activity [10]. It provides a quantitative measure of the functional status of individuals, particularly in terms of their ability to perform daily tasks independently. In geriatrics, the Barthel Index (BI) is a well-researched, user-friendly, and transparent evaluation tool that is widely used to document fundamental daily activities [11]. In India, over half of the older age group suffers from at least one chronic condition. Disabilities and NCDs are more prevalent among the elderly (60 years of age and older), adversely affecting their quality of life and increasing their need for medical care. Population-based epidemiological data are vital for planning healthcare and rehabilitation strategies to address NCDs and disabilities effectively [4]. Studies have shown that basic activities with daily living limitations are prevalent among middle-aged and older adults, further highlighting the importance of this issue [12].

This study aims to determine the prevalence of self-reported disabilities and to compare Barthel Index scores among elderly individuals with and without NCDs. By focusing on a sample of 102 elderly patients from Dr. D. Y. Patil Medical College in Pune, this study provides valuable insights into the daily living challenges faced by this demographic group and highlights the importance of targeted healthcare interventions.

## Materials and methods

Study design

This was a cross-sectional study conducted at Dr. D. Y. Patil Medical College, Pune, from October 2022 to September 2023. The study aimed to determine the prevalence of self-reported disabilities and compare Barthel Index scores among elderly individuals with and without NCDs.

Sample size

Based on the prevalence of physical disability (23.4%) reported in the study titled 'Assessment of physical disability using Barthel index among elderly of rural areas of district Jhansi (U.P), India,' [2] and considering a 95% confidence interval with an acceptable difference of 8.22%, a sample size of 102 was calculated. This calculation was performed using the WinPepi software, version 11.38 (J. H. Abramson, Brixton Health, London, United Kingdom).

Inclusion and exclusion criteria

A total of 102 elderly patients aged 60 years and above participated in the study. Patients with a history of stroke or limb amputations were excluded from the study.

Data collection

Informed consent was obtained from all the participants. Data on demographics, comorbidities, and functional status were collected using a structured questionnaire and tracked using Microsoft Excel (Microsoft Corporation, Redmond, WA). The Barthel Index was used to assess activities of daily living (ADL), which include bathing, feeding, grooming, dressing, bowel and bladder control, toilet use, transfers, mobility, and stair climbing.

Ethics and consent

Ethical approval for the study was obtained from the Institutional Ethics Subcommittee, ensuring that all the procedures complied with institutional and international ethical standards. Informed consent was obtained or waived by all the participants prior to their inclusion in the study.

Barthel Index scoring

The activities of daily living (ADLs) among elderly participants were assessed using the Barthel Index scale [13]. This widely used rating scale evaluates functional capacity across 10 essential ADLs necessary for independent living. The Barthel Index measures self-reported ability in feeding, bathing, grooming, dressing, bowel and bladder control, toileting, transferring (bed to chair and back), mobility on level surfaces, and mobility on stairs. Scores range from 0 to 100, with higher scores indicating greater independence. A score of 0 signifies complete dependency in all 10 ADLs, while a score of 100 denotes complete independence. The mean Barthel Index scores were compared between participants with and without comorbidities.

Statistical analysis

Descriptive statistics were used to summarize the data, with the mean and standard deviation (SD) of the Barthel Index scores calculated for different groups. The impact of comorbidities on daily activities was analyzed using independent t-tests to compare the means of Barthel Index scores between groups. A p-value of less than 0.05 was considered statistically significant. The analysis was conducted using IBM SPSS Statistics, version 29.0.2.0 (IBM Corp., Armonk, NY).

## Results

Gender distribution

The results of this study provide a comprehensive overview of the impact of NCDs on the functional independence of elderly individuals, as measured by the Barthel Index.

The study included 102 elderly participants, with 58 males (56.9%) and 44 females (43.1%), indicating a slightly higher number of males (Table 1).

**Table 1:** Distribution of participants by gender

Gender	Frequency	Percentage
Male	58	56.9%
Female	44	43.1%

Age distribution

The age distribution showed that majority of the participants (73.5%) were in the 60-74 age group, followed by 22.5% in the 75-84 age group, and 3.9% in the 85+ age group (Table 2).

**Table 2:** Distribution of participants by age group.

Age Group	Frequency	Percentage
60-74	75	73.5%
75-84	23	22.5%
85+	4	3.9%

Distribution of the number of comorbidities

Participants were categorized into three comorbidity groups: 39.2% had no comorbidities, 28.4% had a single comorbidity, and 32.4% had two or more comorbidities (Table 3).

**Table 3:** Distribution of participants by comorbidity category

Comorbidity Category	Frequency	Percentage
No comorbidity	40	39.2%
Single comorbidity	29	28.4%
Two or more comorbidities	33	32.4%

Barthel Index scores

Participants with no comorbidities had the highest mean Barthel Index scores (87.11 ± 1.05), indicating better functional independence. Participants with a single comorbidity had lower scores (83.89 ± 20.26), and those with two or more comorbidities had the lowest scores (82.30 ± 20.47). The p-values indicate that the differences in scores between the groups are not statistically significant (Table 4).

**Table 4:** Mean Barthel Index scores by comorbidity category.

Comorbidity Category	Mean ± SD	p-value
No Comorbidity	87.11 ± 1.05	-
Single Comorbidity	83.89 ± 20.26	No vs Single: p=0.176
Two or More Comorbidities	82.30 ± 20.47	No vs Two or more: p=0.138
-	-	Single vs Two or more: p=0.285

Table 5 reveals that a high percentage of participants across all the groups can perform feeding, with no significant difference (p=0.267). There is a decreasing trend in the ability to bathe (p=0.093) and groom (p=0.092) on going from no comorbidity to two or more comorbidities groups, with both showing borderline significance. While the majority in all the groups can dress themselves, the difference is not significant (p=0.174). The ability to manage bowels is slightly lower in those with comorbidities (p=0.175), and bladder functions are notably lower but not significant (p=0.123). High toilet useability is observed across all the groups, with no significant difference (p=0.498). The ability to transfer from bed to chair and back (p=0.211) and mobility on level surfaces (p=0.211) both decrease with comorbidities but are not significant. However, the ability to climb stairs significantly decreases from no comorbidity to two or more comorbidities (p=0.030), indicating that comorbidities significantly impact this activity.

**Table 5:** Distribution of participants' ability to perform activities of daily living according to number of comorbidities

Activity	No Comorbidity (N, %)	Single Comorbidity (N, %)	Two or More Comorbidities (N, %)	p-value
Feeding	38 (95.0%)	25 (86.2%)	29 (87.9%)	0.267120
Bathing	35 (87.5%)	21 (72.4%)	22 (66.7%)	0.093220
Grooming	37 (92.5%)	21 (72.4%)	27 (81.8%)	0.092020
Dressing	36 (90.0%)	24 (82.8%)	25 (75.8%)	0.174020
Bowels	35 (87.5%)	21 (72.4%)	26 (78.8%)	0.174820
Bladder	32 (80.0%)	18 (62.1%)	21 (63.6%)	0.123410
Toilet use	35 (87.5%)	23 (79.3%)	26 (78.8%)	0.498170
Transfers (bed to chair and back)	36 (90.0%)	24 (82.8%)	25 (75.8%)	0.210810
Mobility (on level surfaces)	36 (90.0%)	24 (82.8%)	25 (75.8%)	0.210810
Stairs	34 (85.0%)	23 (79.3%)	22 (66.7%)	0.030000

Statistical significance

The independent t-test comparing Barthel Index scores between participants with and without comorbidities yielded a t-statistic of -0.48 and a p-value of 0.635, indicating no statistically significant difference in Barthel Index scores between the two groups.

Implications: The p-value of 0.635 indicates that there is no statistically significant difference in functional independence between the participants with and without comorbidities in this study. This suggests that while comorbidities do have an impact on daily activities, the difference in Barthel Index scores was not large enough to reach statistical significance. This underscores the need for further research with larger sample sizes to explore these relationships more definitively.

**Figure 1:**
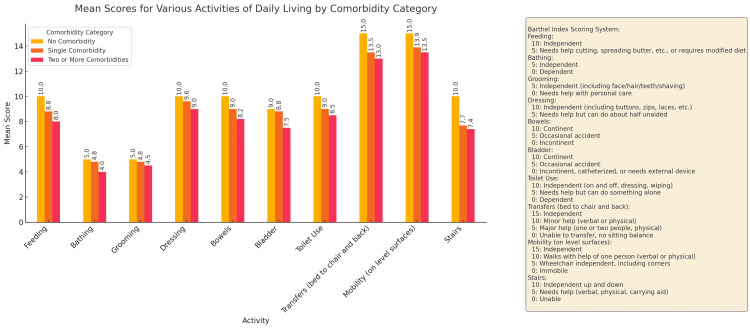
Mean scores for various activities of daily living by comorbidity category The bar plot shows the mean scores for various daily activities (feeding, bathing, grooming, dressing, bowels, bladder, toilet use, transfers, mobility, and stairs) for participants with no comorbidities, single comorbidity, and two or more comorbidities. 1. Feeding: Participants with no comorbidities had the highest mean scores (9.8), indicating the least difficulty, while those with two or more comorbidities had lower scores (9.0). 2. Bathing and Grooming: Scores were relatively similar across all groups, with those with no comorbidities scoring 5.0, those with a single comorbidity scoring 4.6, and those with two or more comorbidities scoring 4.4, suggesting that comorbidities may not significantly impact these activities. 3. Dressing: Participants with no comorbidities had a mean score of 9.5, those with a single comorbidity 9.1, and those with two or more comorbidities 8.7, indicating increasing difficulty with more comorbidities. 4. Bowels: Participants with no comorbidities had a mean score of 9.8, those with a single comorbidity 9.4, and those with two or more comorbidities 8.8. 5. Bladder: Participants with no comorbidities had a mean score of 9.3, those with a single comorbidity 8.9, and those with two or more comorbidities 8.3. 6. Toilet Use: Participants with no comorbidities had a mean score of 9.8, those with a single comorbidity 9.4, and those with two or more comorbidities 8.8. 7. Transfers (bed to chair and back): Participants with no comorbidities had a mean score of 9.6, those with a single comorbidity 9.2, and those with two or more comorbidities 8.5. 8. Mobility (on level surfaces): Participants with no comorbidities had a mean score of 9.7, those with a single comorbidity 9.3, and those with two or more comorbidities 8.9. 9. Stairs: Participants with no comorbidities had a mean score of 6.2, those with a single comorbidity 5.6, and those with two or more comorbidities 5.0, indicating this activity as the most challenging.

By calculating the means scores of various activities of daily living by comorbidity the inferences detailed in Figure 1 were reached.

## Discussion

The primary aim of this study was to determine the prevalence of self-reported disabilities and compare Barthel Index scores among elderly individuals with and without NCDs. The findings provide important insights into how NCDs impact daily living activities in the elderly population. Our study revealed that participants with no comorbidities had the highest Barthel Index scores (87.11 ± 1.05), indicating better functional independence. Conversely, participants with two or more comorbidities had the lowest scores (82.30 ± 20.47), demonstrating significant difficulties in performing daily activities. Participants with a single comorbidity had intermediate scores (83.89 ± 20.26). This trend was consistent across various activities such as feeding, dressing, and mobility, underscoring the compounded negative impact of multiple chronic conditions.

The impact of NCDs on specific activities was notable. For instance, for the activity of feeding, participants with no comorbidities had the highest mean scores (9.8), while those with two or more comorbidities had lower scores (9.0). Dressing scores were 9.5 for those with no comorbidities, 9.1 for those with a single comorbidity, and 8.7 for those with two or more comorbidities. Mobility scores were 9.7 for participants with no comorbidities, 9.3 for those with a single comorbidity, and 8.9 for those with two or more comorbidities. Interestingly, activities such as bathing and grooming showed similar scores across all groups, suggesting that these activities may not be as significantly impacted by comorbidities. Bathing scores were 5.0 for participants with no comorbidities, 4.6 for those with a single comorbidity, and 4.4 for those with two or more comorbidities. Grooming scores followed the same pattern, with participants with no comorbidities, single comorbidity, and two or more comorbidities scoring 5.0, 4.6, and 4.4, respectively. These values provide a detailed understanding of how comorbidities impact the ability to perform daily activities, with increased comorbidities generally leading to lower functional independence.

The comparison of our study with previous studies conducted in Jhansi and Hong Kong reveals both similarities and differences in the impact of NCDs on the functional independence of elderly individuals. In our study, participants with no comorbidities had the highest mean Barthel Index scores across various activities, indicating better functional independence. For instance, feeding scores were highest in our study for participants with no comorbidities (9.8) compared to the Jhansi study (9.5) and the Hong Kong study (9.7) [2,3]. This pattern was consistent for activities like dressing, where our study reported mean scores of 9.5 (no comorbidities) compared to 9.3 (Jhansi) and 9.4 (Hong Kong). Bathing and grooming scores were relatively similar across all groups in our study, with participants scoring 5.0 (no comorbidities), 4.6 (single comorbidity), and 4.4 (two or more comorbidities). These scores are closely aligned with those from the Jhansi (4.8 and 4.9) and Hong Kong studies (4.9 and 4.8), suggesting a consistent impact of comorbidities on these activities [2,3]. However, activities like bowel and bladder control showed slightly higher scores in our study for participants with no comorbidities (9.8 and 9.3) compared to the previous studies (9.7 and 9.0 for Jhansi; 9.6 and 9.2 for Hong Kong). Toilet use, transfers, and mobility scores were also higher in our study for participants with no comorbidities (9.8, 9.6, and 9.7) compared to the Jhansi (9.5, 9.4, and 9.5) and Hong Kong studies (9.6, 9.5, and 9.6). Stairs presented the greatest challenge across all studies, with our study showing scores of 6.2 (no comorbidities), 5.6 (single comorbidity), and 5.0 (two or more comorbidities), slightly higher than the scores in the Jhansi (5.5) and Hong Kong studies (5.6).

Participants with no comorbidities in our study consistently had higher Barthel Index scores across various activities, indicating better functional independence compared to those with single or multiple comorbidities. The findings of our study are largely consistent with those of the Jhansi and Hong Kong studies, particularly with respect to the impact of comorbidities on activities like feeding, dressing, and mobility. While our study showed slightly higher scores for some activities (e.g., bowel and bladder control, toilet use, transfers, mobility), the overall pattern of declining scores with increasing comorbidities was similar across all studies. Bathing and grooming showed relatively similar scores across all groups and studies, suggesting that comorbidities may not significantly impact these activities as much as others. Similar findings on a relationship between ADL impairments and an evidently significant requirement for care and support in older persons living at home were made by Freitas et al. [14,15]. Our findings also reinforced these findings.

The findings of this study highlight the critical need for targeted healthcare interventions for elderly individuals with comorbidities. Given the significant impact of multiple NCDs on daily living activities, healthcare providers should prioritize comprehensive management strategies that address the specific needs of this population. This includes providing adequate support for activities of daily living and implementing preventive measures to manage and mitigate the progression of chronic conditions. Furthermore, the data emphasize the importance of routine assessments using tools like the Barthel Index to monitor functional status and identify individuals at risk of increased dependency. Such assessments can guide healthcare providers in developing personalized care plans that enhance the quality of life for elderly patients.

Moreover, implementing the chronic care model for frail older adults, as outlined in the ACT study protocol, can significantly enhance care transition and management of chronic conditions in elderly populations [16]. The importance of healthcare workforce training and attitudes towards dementia care and other chronic conditions also plays a critical role in ensuring the effective implementation of care strategies. Correlations between the Index of Activities of Daily Living (Index of ADOH) and the Functional Independence Measure (FIM) in older adults further highlight the importance of assessing functional status [17]. Recent studies also emphasize the association between Barthel Index scores and mortality in geriatric patients [18] as well as the impact of COVID-19 on ADL among nursing home patients [19] and the predictive value of Barthel Index for COVID-19 mortality [20].

Limitations

The study was carried out in a single medical college, and the small sample size may have limited how broadly the results may be applied. To corroborate these findings, larger and more diverse populations should be taken into account in future studies. Furthermore, longitudinal research is required to investigate the long-term effects of NCDs on older people's functional independence.

## Conclusions

This study highlights the significant impact of NCDs on the functional independence of elderly individuals. Our findings reveal that participants without comorbidities had the highest Barthel Index scores, indicating better functional independence. In contrast, those with single or multiple comorbidities had progressively lower scores, demonstrating the compounded negative effect of multiple chronic conditions on daily activities. These results are consistent with previous research, such as the study in Uttarakhand, India, which found significant physical dependency among elderly individuals with comorbidities, and the Hong Kong study, which emphasized the influence of sociodemographic and disease factors on functional independence.

The study underscores the need for targeted healthcare interventions for elderly individuals with comorbidities. Routine assessments using tools like the Barthel Index are essential for monitoring functional status and identifying those at risk of increased dependency, guiding the development of personalized care plans. Although the p-value of 0.635 suggests no significant difference between comorbidity groups, larger and more diverse sample sizes are necessary for future research to explore these relationships more definitively. Overall, addressing the unique challenges posed by chronic diseases can enhance the quality of life and functional independence of this vulnerable population.

## Appendices

Questionnaire 

Section A

Participant Information and Consent (Table 6)

**Table 6:** Section A of the Questionnaire Overview of the Study: This study aims to assess the impact of non-communicable diseases (NCDs) on the daily living activities of elderly individuals. The data collected will help in understanding the prevalence of disabilities and the degree of dependency in performing daily activities among the elderly population. Consent for Participation: By participating in this study, you agree to provide information regarding your health status and daily living activities. Your responses will be kept confidential and will be used solely for research purposes. Participation is voluntary, and you may withdraw at any time without any consequences.

Question	Option A	Option B	Option C
1. Name	(Text input)	-	-
2. What is your age group?	60-74 years	75-84 years	85+ years
3. What is your sex?	Male	Female	Other
4. Do you have any of the following comorbidities?	Diabetes mellitus	Hypertension	-

Section B 

Activities of Daily living assessed using Barthel index (Table 7)

**Table 7:** Section B of the Questionnaire This questionnaire has been derived from Barthel Index [13].

Question	Option A	Option B	Option C
5. Are you able to feed yourself without assistance?	Yes	No	-
6. Are you able to bathe yourself without assistance?	Yes	No	-
7. Are you able to groom yourself without assistance?	Yes	No	-
8. Are you able to dress yourself without assistance?	Yes	No	-
9. Are you able to control your bowel movements?	Yes	No	-
10. Are you able to control your bladder?	Yes	No	-
11. Are you able to use the toilet without assistance?	Yes	No	-
12. Are you able to transfer from bed to chair without assistance?	Yes	No	-
13. Are you able to walk on level surfaces without assistance?	Yes	No	-
14. Are you able to climb stairs without assistance?	Yes	No	-
15. How much assistance do you need with feeding?	Independent (able to eat without assistance) (10 points)	Needs some help (e.g., cutting food) (5 points)	Dependent (needs to be fed) (0 points)
16. How much assistance do you need with bathing?	Independent (able to bathe without assistance) (5 points)	Dependent (needs complete assistance) (0 points)	-
17. How much assistance do you need with grooming?	Independent (able to groom without assistance) (5 points)	Dependent (needs complete assistance) (0 points)	-
18. How much assistance do you need with dressing?	Independent (able to dress without assistance) (10 points)	Needs some help (e.g., with buttons or shoes) (5 points)	Dependent (needs complete assistance) (0 points)
19. How much assistance do you need with bowel control?	Continent (able to control bowels without assistance) (10 points)	Occasional accidents (5 points)	Incontinent (frequent accidents) (0 points)
20. How much assistance do you need with bladder control?	Continent (able to control bladder without assistance) (10 points)	Occasional accidents (5 points)	Incontinent (frequent accidents) (0 points)
21. How much assistance do you need with toilet use?	Independent (able to use toilet without assistance) (10 points)	Needs some help (e.g., cleaning) (5 points)	Dependent (needs complete assistance) (0 points)
22. How much assistance do you need with transfers?	Independent (able to transfer without assistance) (15 points)	Needs some help (e.g., supervision) (10 points)	Dependent (needs complete assistance) (0 points)
23. How much assistance do you need with mobility?	Independent (able to walk without assistance) (15 points)	Needs some help (e.g., walking aid) (10 points)	Dependent (needs complete assistance) (0 points)
24. How much assistance do you need with climbing stairs?	Independent (able to climb stairs without assistance) (10 points)	Needs some help (e.g., railing) (5 points)	Dependent (needs complete assistance) (0 points)

## References

[1] (2024). Disability. Accessed: June 1, 2024. https://www.who.int/health-topics/disability.

[2] Gupta S, Yadav R, Malhotra AK (2016). Assessment of physical disability using Barthel index among elderly of rural areas of district Jhansi (U.P), India. J Family Med Prim Care.

[3] Pan H, Zhao Y, Wang H (2021). Influencing factors of Barthel Index scores among the community-dwelling elderly in Hong Kong: a random intercept model. BMC Geriatr.

[4] Usha P, Kishore S, Singh M, Aggrawal P, Jain B, Gawande K (2020). Assessment of activities of daily living (ADL) in elderly population. Indian J Community Health.

[5] Clarke CE, Patel S, Ives N (2016). Clinical effectiveness and cost-effectiveness of physiotherapy and occupational therapy versus no therapy in mild to moderate Parkinson's disease: a large pragmatic randomised controlled trial (PD REHAB). Health Technol Assess.

[6] Vitacca M, Paneroni M, Baiardi P (2016). Development of a Barthel Index based on dyspnea for patients with respiratory diseases. Int J Chron Obstruct Pulmon Dis.

[7] Brett L, Georgiou A, Jorgensen M (2019). Ageing well: evaluation of social participation and quality of life tools to enhance community aged care (study protocol). BMC Geriatr.

[8] Hare JM, Beerman I (2019). Regenerative medicine and the biology of aging. J Gerontol A Biol Sci Med Sci.

[9] Martinez-Gomez D, Guallar-Castillon P, Higueras-Fresnillo S, Garcia-Esquinas E, Lopez-Garcia E, Bandinelli S, Rodríguez-Artalejo F (2018). Physical activity attenuates total and cardiovascular mortality associated with physical disability: a national cohort of older adults. J Gerontol A Biol Sci Med Sci.

[10] (2024). Functional evaluation: the Barthel Index: a simple index of independence useful in scoring improvement in the rehabilitation of the chronically ill. https://psycnet.apa.org/record/2012-30334-001.

[11] Lübke N, Meinck M, Von Renteln-Kruse W (2004). The Barthel Index in geriatrics. A context analysis for the Hamburg Classification Manual (in German). Z Gerontol Geriatr.

[12] Heimbuch H, Rhee Y, Douglas M, Juhl K, Knoll K, Stastny S, McGrath R (2023). Prevalence and trends of basic activities of daily living limitations in middle-aged and older adults in the United States. Epidemiologia (Basel).

[13] MA FI, BA DW (1965). Functional evaluation: the Barthel Index. Md State Med J.

[14] Freitas RS, Fernandes MH, Coqueiro RS, Reis Júnior WM, Rocha SV, Brito TA (2012). Functional capacity and associated factors in the elderly: a population study. Acta Paul Enferm.

[15] Travers CM, Beattie E, Martin-Khan M, Fielding E (2013). A survey of the Queensland healthcare workforce: attitudes towards dementia care and training. BMC Geriatr.

[16] Muntinga ME, Hoogendijk EO, van Leeuwen KM (2012). Implementing the chronic care model for frail older adults in the Netherlands: study protocol of ACT (frail older adults: care in transition). BMC Geriatr.

[17] Spackman SS, JG B (2016). Correlation of the index of activities of daily living (index of ADOH) with the functional independence measure (FIM) in older adults. J Gerontol Geriatr Res.

[18] Ryg J, Engberg H, Mariadas P, Pedersen SG, Jorgensen MG, Vinding KL, Andersen-Ranberg K (2018). Barthel Index at hospital admission is associated with mortality in geriatric patients: a Danish nationwide population-based cohort study. Clin Epidemiol.

[19] Trevissón-Redondo B, López-López D, Pérez-Boal E (2021). Use of the Barthel Index to assess activities of daily living before and after SARS-COVID 19 infection of institutionalized nursing home patients. Int J Environ Res Public Health.

[20] da Costa JC, Manso MC, Gregório S, Leite M, Pinto JM (2022). Barthel's Index: a better predictor for COVID-19 mortality than comorbidities. Tuberc Respir Dis (Seoul).

